# Nutritional assessment system integrating semantic segmentation and point cloud modeling techniques

**DOI:** 10.1038/s41598-025-23096-7

**Published:** 2025-11-10

**Authors:** Chin-Hsuan Lin, Jung-Tang Huang

**Affiliations:** Institute of mechatronic engineering, National Taipei University of Technology, Taipei, Taiwan

**Keywords:** Edge device, Depth camera, Semantic segmentation, Point cloud modeling, Nutritional assessment, Engineering, Mathematics and computing, Quality of life

## Abstract

Balanced nutrition plays a vital role in preventing chronic diseases. We propose a home-based monitoring system that integrates an Nvidia Jetson AGX Xavier embedded device with an Intel RealSense D435 depth camera mounted vertically above the dining table. The system collects data every minute during meals, capturing point clouds with RGB information. Deep-learning object detection models identify and track multiple food items, ensuring each item is recognized independently. Point clouds are aligned using Random Sample Consensus (RANSAC) and Iterative Closest Point (ICP), while Density-Based Spatial Clustering of Applications with Noise (DBSCAN) distinguishes the table, plate, and food. The system records temporal changes in meal portions and reconstructs food point clouds to estimate volume. Each estimate is linked to the corresponding food category predicted by the semantic segmentation model and combined with nutritional values. All data are uploaded to the cloud, enabling user analysis along with preliminary assessments of total nutrient intake and eating speed. By integrating these components, the system provides accurate records of meal volume and composition, supporting comprehensive evaluations of home-based nutrition over a three-month period.

## Introduction

 In modern society, advances in medical technology and rising health awareness have made population aging a global trend. As age increases, older adults face multiple health challenges, with nutrition-related problems being especially prominent. Poor dietary habits can lead to malnutrition, sarcopenia, obesity, hypertension, cardiovascular disease, diabetes, chronic kidney disease, and other conditions^1,2^. Therefore, effective monitoring and management of older adults’ diets has become an urgent need.

Traditional dietary management methods rely mainly on self-reporting, which is prone to memory bias and subjective error, often leading to poor nutritional outcomes. Although various diet-tracking applications and image recognition tools are available^3^, they typically require complex operations or manual input, such as weighing food or entering dish information^4^. These demands are burdensome for older adults and reduce the likelihood of long-term use^5^.

Moreover, existing dietary databases have limited coverage. With millions of recipes and diverse home-cooking styles worldwide, building a comprehensive database manually is impractical^6^. In home-cooking environments in particular, dishes with the same name may vary greatly due to differences in ingredients and proportions.

To address these challenges, this study develops an automated nutritional assessment system that can accurately identify dietary content and perform nutritional analysis while considering user compliance and the diversity of food. Our approach integrates depth cameras with an edge computing platform, combining object recognition, semantic segmentation, and point cloud modeling to achieve automatic dietary identification and nutritional value calculation. Such design choices align with recent analyses of machine learning approaches and deployment trends on edge AI platforms^7^.

The main contributions of this study include: (1) developing an automated nutritional assessment system that integrates edge computing devices and depth cameras, and providing a website for healthcare professionals to access dietary records and explore diet–disease relationships; (2) proposing data collection and processing methods for real dining environments, with a focus on assessing eating speed; (3) placing a camera at a fixed height above the dining table, with its view restricted to the table area, and using a Bluetooth module connected to an IMU device worn on the user’s chest to automatically activate or deactivate the camera when approaching the dining area, thereby reducing privacy concerns while enabling automatic recording of eating, medication intake, or drinking events; (4) experimentally verifying the system’s effectiveness in improving user compliance while maintaining accuracy; (5) conducting dietary trials with one two-person household over two months, involving two participants over 63 years of age.; and (6) This paper proposes methodological innovations for dynamic food monitoring. Unlike traditional approaches that only estimate static volumes, our method captures continuous changes in food volume across multiple time points. A distance-threshold filtering strategy enables targeted extraction of surface changes caused by food removal, while a dynamic Z-axis origin correction ensures precise alignment of sequential point clouds for accurate volume computation. Furthermore, by combining localized surface projection with Delaunay triangulation, the system significantly reduces computational cost compared with global mesh reconstruction, improving efficiency and supporting near real-time applications.

The experimental results indicate that, compared with traditional dietary recording methods, the system provides a near-zero-intervention solution despite an error rate of 10%–20% (mainly due to nutritional database limitations and the complexity of food stacking). This outcome offers valuable support for smart home care and is expected to significantly enhance nutritional management and overall health in older adults and individuals with chronic diseases.

## Methods

This study followed the principles of the Declaration of Helsinki and was approved by the Institutional Review Board of MacKay Memorial Hospital (IRB No. 20MMHIS504e). Written informed consent was obtained from all participants or their legal guardians.

### Related works

For automated nutritional assessment, image-based methods are commonly used to identify food items. Approaches include stereo vision, time-of-flight (TOF), and structured light devices for capturing 3D food models. Among 2D image-based approaches, Konstantakopoulos et al. applied stereo vision for volume estimation^8,9^. Their smartphone-based algorithm paired pre-processed images using the RANSAC algorithm^10^ to obtain camera rotation and translation matrices. Stereo matching was then performed to compute pixel disparities in rectified images, generating a dense depth map. A weighted least squares filter refined the disparity map by aligning edges and propagating values across confidence regions. The depth map was converted into a point cloud, followed by Delaunay triangulation^11^ to form a triangular mesh surface, and volume was estimated using a convex hull method.

In depth sensing, Yoshikazu Ando et al. developed a calorie estimation application using the iPhone XS LiDAR sensor^12^. Food items were segmented into slender rectangular parallelepipeds, and their volumes were calculated by multiplying segment depth and area. Summing these values yielded the total volume, similar to surface integration.

Food recognition remains challenging due to the diversity and complexity of food types. Numerous approaches have been proposed, with convolutional neural networks (CNNs) being the most widely applied in image-based deep learning. Popular models include YOLO, ResNet, Inception, and EfficientNet. Among them, YOLO is particularly favored for its strong real-time detection capability, enabling simultaneous identification of multiple objects in a single forward pass. Its efficiency and speed make it well suited for applications that demand rapid recognition, such as autonomous driving and surveillance.

In terms of dataset construction, Seon-Joo Park et al. collected more than 4,000 Korean food images through web scraping and photography, and expanded the dataset to 92,000 images using data augmentation. These images were classified into 23 categories for training a deep convolutional neural network (DCNN)^13^. Because publicly available Korean food datasets are limited, the authors relied on augmentation and image processing to overcome data scarcity and improve model performance. Augmentation methods generated new images from single samples, while image processing techniques enhanced quality and reduced redundancy. Adjustments such as random changes in contrast, brightness, sharpness, and color increased variability, ultimately expanding the dataset to 92,000 images.

In recent years, visual-based dietary assessment (VBDA) systems have advanced considerably, typically comprising three stages: food image analysis, portion estimation, and nutritional content inference. Baban A. Erep proposed the mid-DeepLabv3 + model^14^, an adaptation of DeepLabv3 + with a ResNet50 backbone^15^. This semantic segmentation model incorporates the SimAM self-attention mechanism to calculate neuron importance weights without increasing network parameters. The architecture also introduces an additional decoder middle layer and applies SimAM after each backbone feature layer. Furthermore, the author introduced the CamerFood10 dataset for Sub-Saharan African food segmentation, which contains images characterized by inter-class similarity and mixed food components. On this dataset, mid-DeepLabv3 + achieved a mean Intersection over Union (mIoU) of 65.20%, improving by 10.74% over the original DeepLabv3+.

Accurate portion-size estimation is critical for evaluating calories and nutritional values. To obtain reliable food volumes, whether through image-based methods or depth cameras, point cloud processing is essential. In stereo vision, even with CLAHE contrast equalization, stability and accuracy remain lower than those of structured light sensors. Given the lightweight, portable design of our system, we adopted structured light depth cameras for point cloud acquisition, as in^16^.

Our volume calculation approach follows the method described in^8^, where point clouds are converted into triangular meshes for volume computation. For food segmentation, however, we employ the clustering method in^16^ to separate the main object from the background, allowing the DeepLabv3 + model to identify food components more effectively^15^. Unlike traditional methods that only classify food categories, this approach determines the proportion of food within specific areas, providing more detailed recognition results.

### System design

**Fig. 1:**
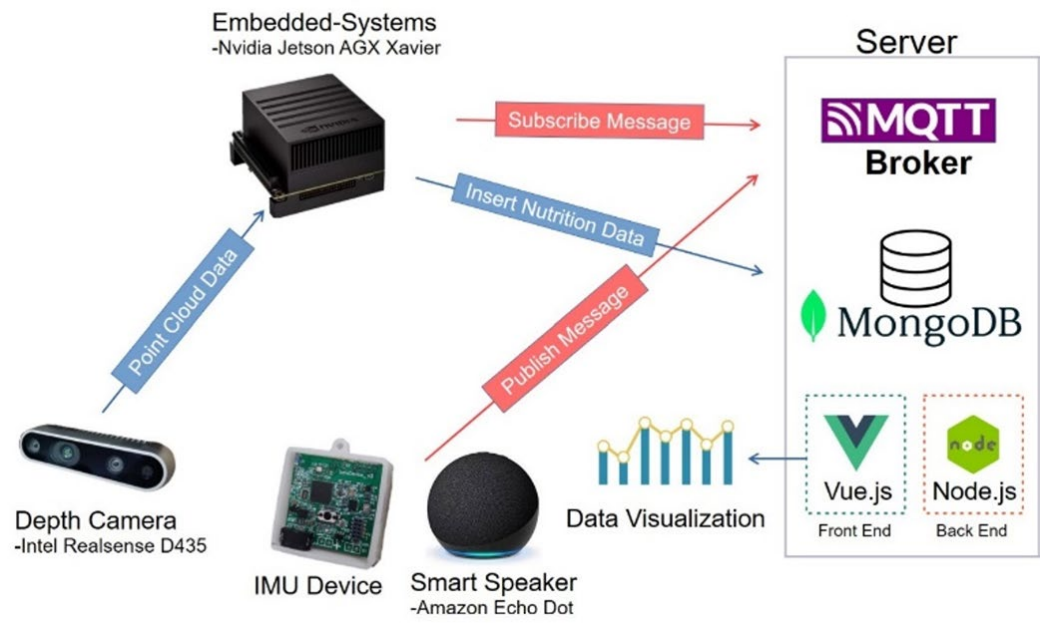
Architecture diagram of the iot nutrition data management and visualization system.

This study establishes a nutritional assessment system using the Nvidia Jetson AGX Xavier embedded platform and the Intel RealSense D435 depth camera to capture point cloud and RGB image data. The food evaluation algorithm is implemented in Python. The system supports voice control via Amazon smart speakers, as well as posture detection and indoor positioning through a chest-worn IMU device and a Beacon developed by our lab^17,18^. Both methods use the MQTT communication protocol to send commands to the embedded platform, enabling the start or stop of meal monitoring, as illustrated in Fig. 1.

Nutritional data are published in real time to a MongoDB non-relational database on the server. The system also features a frontend (Vue.js) and backend that provide a visualization platform with dashboards and related tools for users, family members, and healthcare professionals to monitor and review dietary information.

**Fig. 2:**
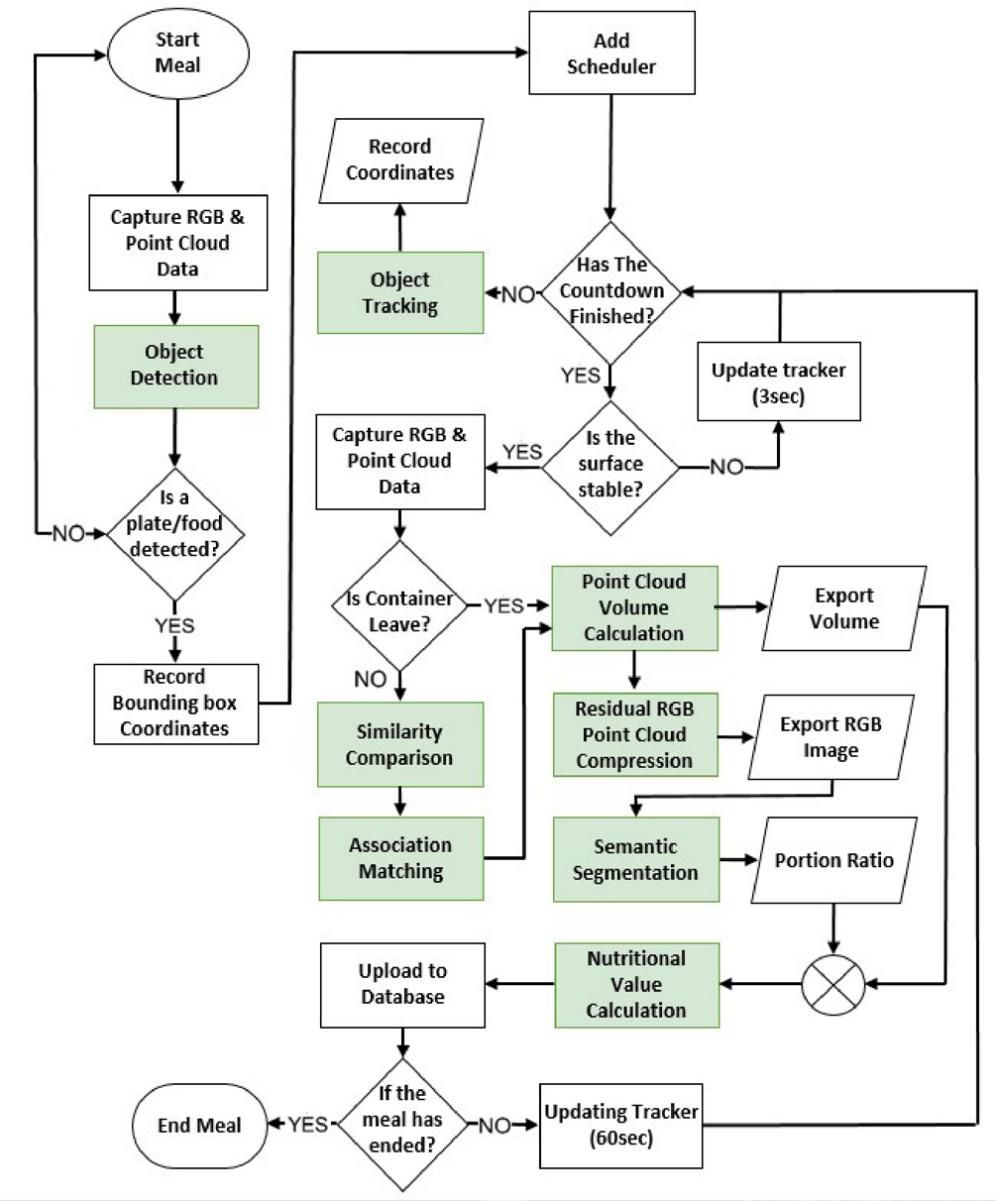
Algorithm flowchart.

The algorithmic workflow developed in this study for real dining scenarios consists of several stages: object recognition, object tracking, similarity comparison, semantic segmentation for food component identification, point cloud volume calculation, compression of residual RGB point clouds, and nutritional value estimation. At the beginning of meal monitoring, the coordinates of the plates on the table are used as initial values and entered into the tracking model. Based on these tracking coordinates, the system estimates the nutritional values of food in different containers, as illustrated in Fig. 2.

The algorithm workflow can be broken down into the following steps:

A. Capture RGB & Point Cloud Data:

The RGB-D camera captures images and point cloud information of the dining table for subsequent object recognition and tracking.

B. Object Recognition and Tracking:

The YOLOv5 deep learning model detects plate locations, and the Minimum Output Sum of Squared Error (MOSSE) filter tracks their positions.

C. Similarity Comparison and Target Association Matching:

Image matching is performed using Transformer feature cosine similarity, plate diameter similarity, and RGB channel similarity to compare the current image with a reference. The Hungarian algorithm then associates historical object locations with current detection results.

D. Point Cloud Volume Calculation and Residual RGB Point Cloud Compression:

Point cloud data are used to calculate food and plate volumes. Segmentation algorithms isolate food-related point clouds, and residual RGB point clouds are compressed into a 2D plane to generate images free from background noise for semantic segmentation.

E. Semantic Segmentation for Food Component Identification:

The DeepLabv3 + model performs semantic segmentation to distinguish food types and proportions, providing input for nutritional value estimation.

F. Nutritional Value Calculation:

Food categories and volumes are matched with nutritional databases to calculate values. This process includes converting food volume to mass and applying the corresponding nutritional data.

### Point cloud modeling & volume estimating

**Fig. 3:**
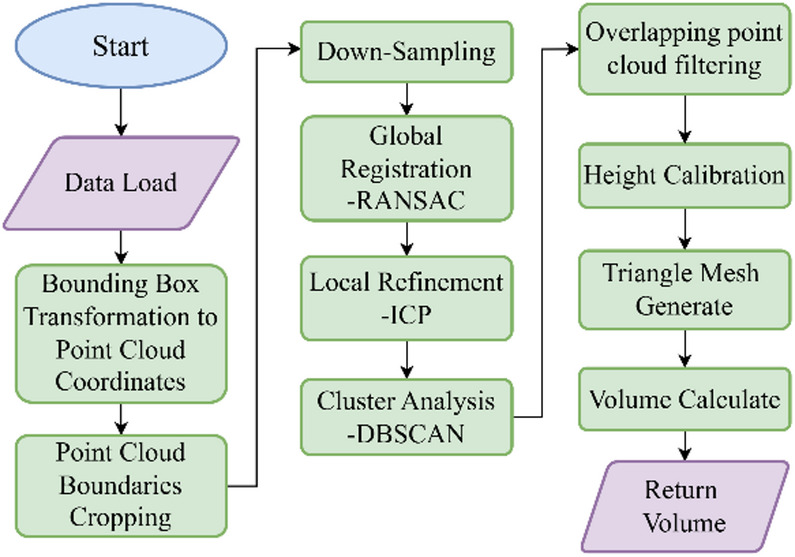
Volume calculation workflow.

To isolate the food point cloud, a series of processing steps are applied to remove background noise, enabling accurate food modeling and mesh-based volume calculation, as illustrated in Fig. 3.

A. ***RGB coordinate projection and point cloud cropping***.
B. For object recognition and tracking, RGB images from the RealSense D435’s built-in camera are used. To locate plate objects in the point cloud, 2D pixel coordinates are projected into 3D space. This process requires the camera’s intrinsic parameters: focal lengths $\:{f}_{x}$​ and $\:{f}_{Y}$​, and principal point coordinates $\:{c}_{x}$​ and $\:{c}_{Y}$​. Equations (1)–(3) convert pixel coordinates and depth values into 3D space coordinates (X, Y,Z), where ($\:u$, $\:v$) are the 2D pixel coordinates and $\:d$ is the depth value at that point.
  1 $$\:X=\left(u-{c}_{x}\right)\cdot\:\frac{d}{{f}_{x}}$$
  2 $$\:Y=\left(v-{c}_{Y}\right)\cdot\:\frac{d}{{f}_{Y}}$$
  3 $$\:Z=d$$

### Point cloud registration

- After downsampling the point cloud, Fast Point Feature Histograms (FPFH) are computed and used as input to the Random Sample Consensus (RANSAC) algorithm^10^. Introduced by Fischler and Bolles in 1981, RANSAC iteratively infers a mathematical model from data that may contain outliers. Its main advantage is robustness against outliers, enabling iterative refinement toward an optimal solution. The workflow involves randomly selecting a subset of $\:k$ points to fit a model, evaluating outliers using least squares, and refining the model until the predefined number of iterations is reached. In global registration, RANSAC provides a rapid method for point cloud alignment and supplies the Iterative Closest Point (ICP) algorithm with an optimal initialization for subsequent localization. Compared with using ICP alone, the combined RANSAC–ICP approach is more efficient and robust.

### Foreground-background filtering.

**Fig. 4:**
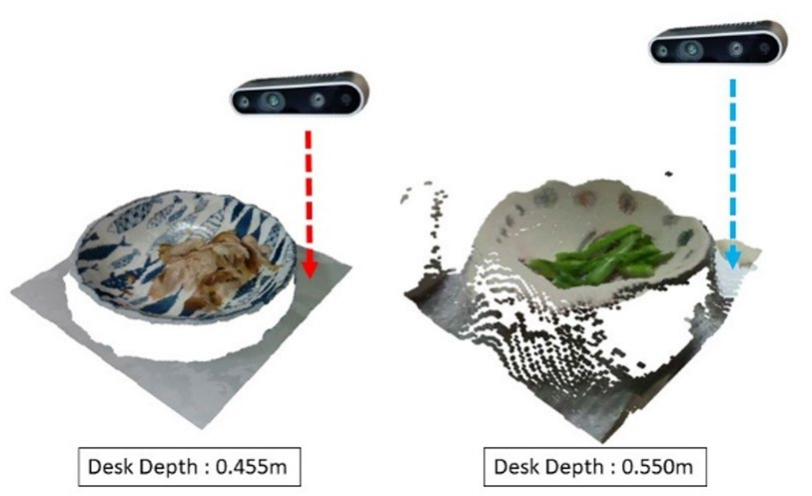
Point Cloud Quality at Different Distances.

- In the collected point cloud, background noise and irrelevant objects often appear alongside the main subject, making foreground–background separation necessary. Clustering analysis is then applied to isolate block-like food items for independent volume calculation. In this study, we use the Density-Based Spatial Clustering of Applications with Noise (DBSCAN) algorithm^19^, which is widely adopted for point cloud segmentation. Proposed by Martin Ester and colleagues, DBSCAN is well suited for spatial data and can identify clusters of arbitrary shapes. The algorithm effectively removes noise and segments distinct entities, improving the accuracy and reliability of both segmentation and subsequent calculations.
- After clustering analysis, separating the foreground from the background is generally straightforward in controlled scenarios. However, practical tests show that clustering alone may lack robustness. This issue arises when depth variation between the foreground and background is minimal, causing point clouds to connect after smoothing. Such effects are often influenced by camera angle, distance, lighting, or surface reflectivity. When the camera is positioned far from the object or the surface reflects light poorly, point cloud density decreases, producing sparse regions. As illustrated in Fig. 4, these conditions may cause the foreground and background to merge, making it difficult to isolate the main subject.

**Fig. 5:**
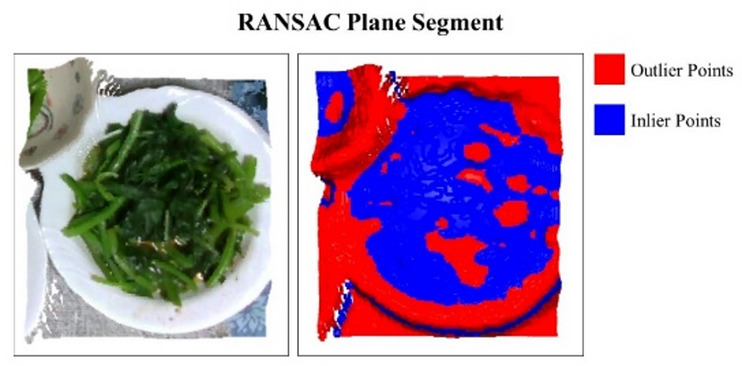
Clustering with RANSAC to separate plate from table plane segmentation.

In typical home dining, three to five dishes are usually placed close together on the table due to space limitations. After point cloud cropping, data from adjacent plates may still appear along the diagonals or edges. Because of this proximity, clustering alone often fails to cleanly segment connected point clouds. To handle such cases, this study combines RANSAC to filter planar point clouds, as illustrated in Fig. 5. The remaining point clouds are then segmented using clustering. To further refine the results, the Root Mean Square (RMS) formula calculates the distance of each cluster from the global point cloud’s center. Clusters within a threshold distance are considered part of the main subject. This approach prevents the exposed flat bottom surface of an empty plate from being misclassified as food once the food has been removed.

**Fig. 6:**
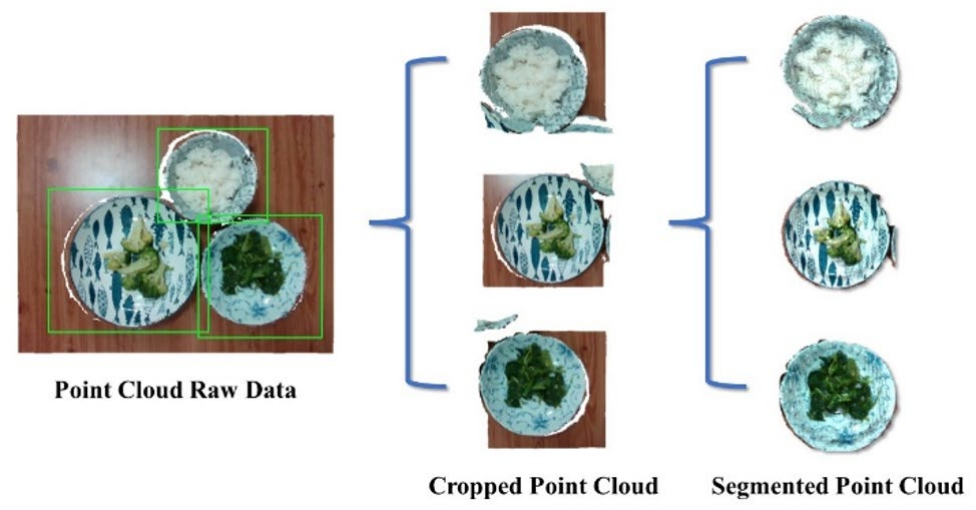
Point cloud segmentation workflow.

When segmenting multiple plates and separating them from the table, as shown in Fig. 6, accurate plate localization is essential. Cropping reduces background noise and improves the success rate of foreground–background separation, which is especially important under sparse point cloud conditions such as those illustrated in Fig. 4. Effective cropping filters out excess background points, enabling more precise segmentation and subsequent processing. This ensures that the segmented point cloud reliably represents the plates’ shape and content.

A. *Point Cloud Mesh Volume Calculation.*

**Fig. 7:**
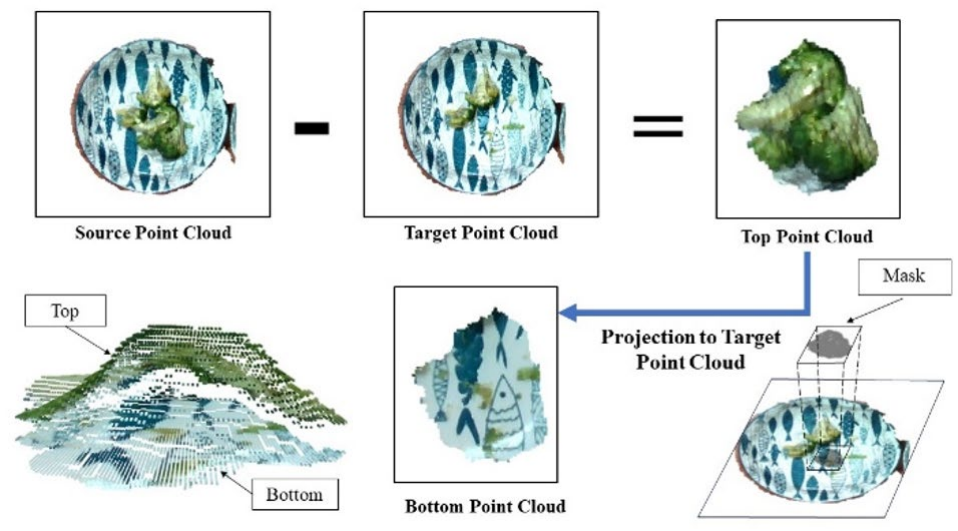
Computation process for changes in food point clouds.

After foreground–background separation, only the plate and connected food point clouds remain. To measure volume changes, the system computes differences between point clouds at different time points. Specifically, the shortest distance from each point in the original point cloud to the target point cloud is calculated. Points exceeding a defined threshold are filtered out, yielding the top point cloud, from which surface features are extracted (Fig. 7).

Subsequently, the surface feature point cloud is projected onto the target point cloud to derive the bottom features, enabling accurate measurement of volume changes during sequential food consumption.

**Fig. 8:**
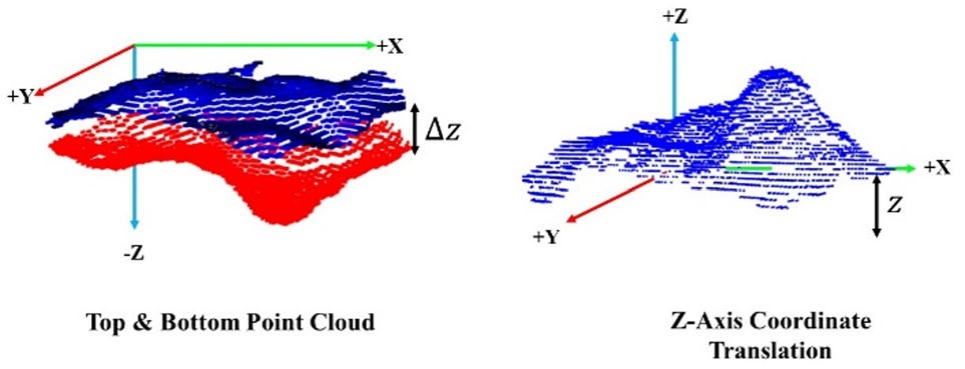
Calibration of the Z-axis origin in point clouds.

In this study, to calculate volume changes caused by food being picked up—particularly when point clouds are captured at intervals consistent with normal eating speed—it is necessary to accurately compare multiple point cloud datasets. Before mesh generation, the coordinates must be adjusted relative to the origin. Because the depth camera provides point clouds with its own origin, volume calculation requires aligning data from two time points ($\:PointClou{d}_{t}$ and $\:PointClou{d}_{t+1}$). These point clouds form the top and bottom of the mesh. The bottom point cloud serves as the reference plane, while the top point cloud is transformed accordingly for volume computation, as illustrated in Fig. 8 for Z-axis origin correction.

Using the relative positions of point cloud coordinates along the Z-axis, a translation is applied. Let a point in the top point cloud be $\:T\left(x,y,z\right)$ and a point in the bottom point cloud be $\:B\left({x}^{{\prime\:}},{y}^{{\prime\:}},{z}^{{\prime\:}}\right)$. When the nearest point $\:B$ to $\:T$ is found in the x–y plane, the coordinates of $\:T$ are translated to obtain a new point $\:{T}_{new}$​. This is expressed in Eq. (4), where $\:x$ and $\:y$ remain unchanged, and the Z-axis adjustment is given by the difference between $\:T$ and $\:B$, as shown in Eq. (5).

4 $$\:{T}_{new}(x,y,\varDelta\:z)$$

5 $$\:\varDelta\:z=z-z{\prime\:}$$

Using the Delaunay triangulation algorithm, the final step generates coordinates that form a triangular mesh. In three-dimensional space, each triangular face is defined by three points, $\:A$, $\:B$, and $\:C$, which specify its positions. These coordinates are expressed in Eqs. (6)-(8).

6 $$\:A=[{X}_{1},{Y}_{1},{Z}_{1}]$$

7 $$\:B=\left[{X}_{2},{Y}_{2},{Z}_{2}\right]$$

8 $$\:C=[{X}_{3},{Y}_{3},{Z}_{3}]$$

**Fig. 9:**
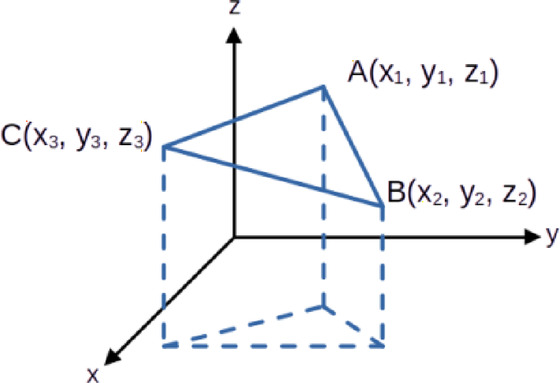
Volume Range of Triangular Mesh Models.

After defining the relationships between the vertices of triangular faces in 3D space, the volume $\:V$ of the truncated triangular prism formed between a triangular face and the reference plane $\:z=0$ is calculated using Eq. (10). The coordinates of the triangular face are shown in Fig. 9, where the dashed lines indicate the projection of the triangular mesh onto the X–Y plane.

9 $$\:V=\frac{1}{6}\left|\left({z}_{1}+{z}_{2}+{z}_{3}\right)\right|\left|\left({x}_{1}{y}_{2}-{x}_{2}{y}_{1}+{x}_{2}{y}_{3}-{x}_{3}{y}_{2}+{x}_{3}{y}_{1}-{x}_{1}{y}_{3}\right)\right|$$

### Ingredient analysis

A. *Semantic Segmentation*.

**Fig. 10:**
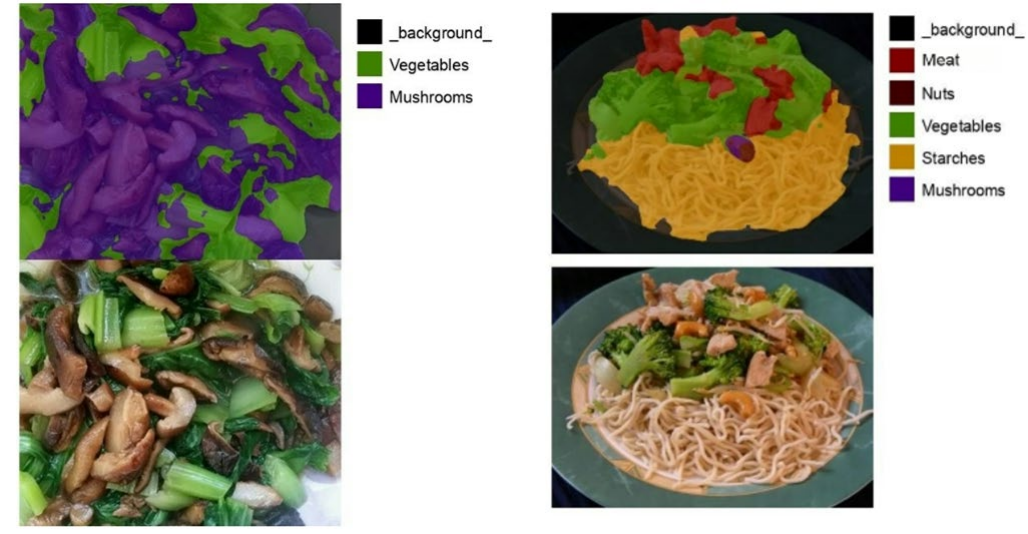
Food semantic segmentation results.

To address food diversity and the varying composition ratios of dishes caused by different cooking methods and flavor preferences, this study applies the semantic segmentation model DeepLab v3 + to RGB images to calculate dish composition ratios. DeepLab v3 + is an improved version of DeepLab v3, incorporating an encoder–decoder structure to reduce the computational cost of high-resolution images and atrous convolution to extract features at multiple scales. These enhancements overcome the limitations of the original model. As shown in Fig. 10, the segmentation results demonstrate that for mixed-type foods, the model can accurately identify masked regions and their proportions, enabling further nutritional analysis.

Recent advances have also highlighted the effectiveness of Transformer-based models in semantic modeling for natural language processing^20^. Inspired by these findings, we adapted similar principles to dietary image analysis, allowing the Vision Transformer to capture both local details and global relationships among food items.

Before calculating nutritional values, the food volume on the plate is estimated using the algorithm proposed in this study. However, most standard methods require multiplying food weight by the nutritional value per unit weight. Thus, the density $\:\varvec{\rho\:}$ of the target food must first be obtained. Moreover, the volume measured by the depth camera does not represent the true food volume but is influenced by the packing density of stacked items^21,22^. Consequently, the final weight is adjusted by multiplying it with a packing density factor $\:\varvec{\mu\:}$, as expressed in Eq. (10).

10 $$\:\varvec{w}\varvec{e}\varvec{i}\varvec{g}\varvec{h}\varvec{t}=\varvec{v}\varvec{o}\varvec{l}\varvec{u}\varvec{m}\varvec{e}\times\:\varvec{\rho\:}\times\:\varvec{\mu\:}$$

Food density $\:\rho\:(g/{cm}^{3})$ for different categories was obtained from the FoodData Central database of the United States Department of Agriculture (USDA). Based on the classifications generated by the semantic segmentation model, average values of density, calories, energy, protein, carbohydrates, fats, and dietary fiber were calculated. Because nutritional values of similar foods may vary across countries and regions, this study also incorporated data from the Food Nutrition Database of the Taiwan Food and Drug Administration (TFDA) to reduce discrepancies. Multiple samples from different food categories were selected, and their average nutritional values were computed. However, because the TFDA database does not provide food density data, USDA values were ultimately adopted as the primary reference.

### Camera trigger

The primary goal of this system is to allow elderly individuals living alone to conduct dietary assessment and record keeping independently. To achieve this, we propose two methods for triggering the meal monitoring process.

A. Smart Speaker.

Through Amazon’s Alexa voice assistant, a customized interaction mode can be established. Using predefined key phrases, users trigger custom logic via Amazon Skills. This service executes code on AWS Lambda to process the SDK and generate the appropriate response.

**Fig. 11:**
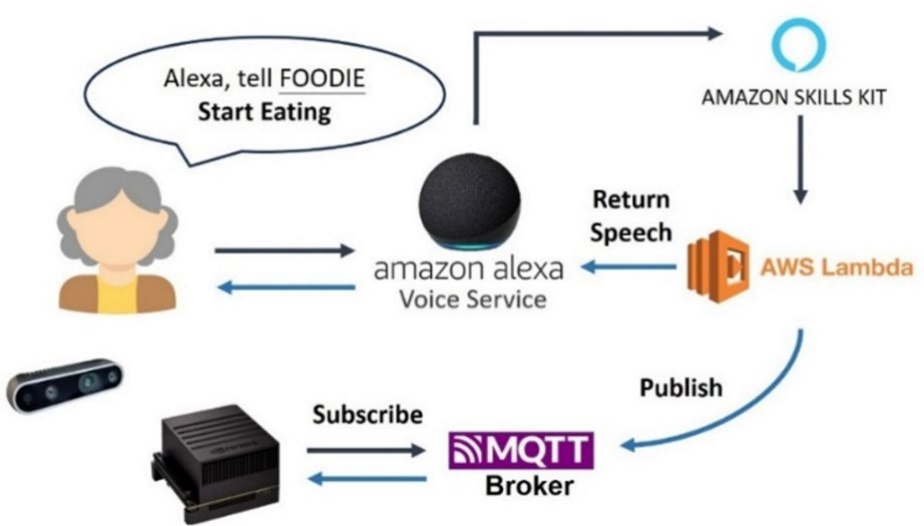
Architecture diagram of alexa skill invocation.

In this study, a custom Amazon Skill named *FOODIE* is used. When the command “Start Eating” is given, the skill publishes an MQTT message. The nutritional assessment system, which subscribes to this message, then begins capturing and recording the user’s meal. The system architecture is illustrated in Fig. 11.

B. IMU Posture and BLE Beacon Indoor Positioning.

**Fig. 12:**
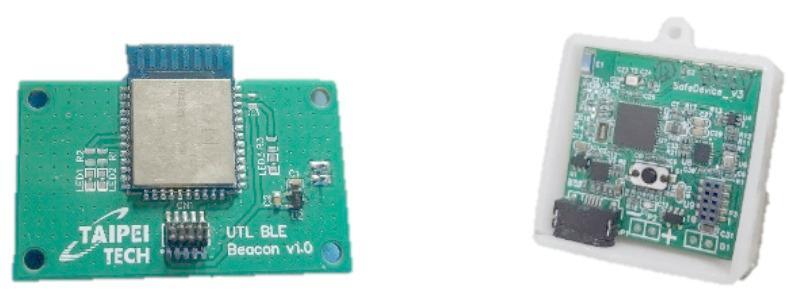
(**a**)BLE Beacon (**b**)IMU Device.

As shown in Fig. 12, the BMX160 nine-axis sensor and BMP280 barometric sensor analyze posture changes using acceleration, angular velocity, magnetic field, and air pressure data. The system recognizes seven postures: sitting, standing, lying on the back, right side, left side, stomach, and walking.

**Fig. 13:**
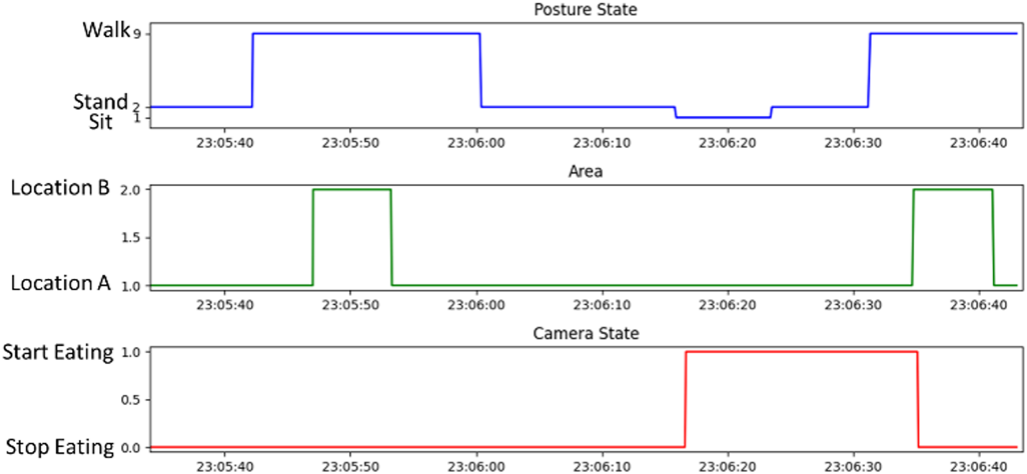
Diagram of Posture Trigger Relationship.

Indoor positioning is determined using BLE Beacons and RSSI signals from the IMU device combined with fingerprint positioning^17,18^. As illustrated in Fig. 13, when the user is seated at the dining table, the system automatically starts meal recording. If the user leaves the table and the Beacon fingerprint updates to another area, recording stops. This approach enables automatic dietary monitoring based on posture and location, eliminating the need for manual intervention.

### Tracking & similarity comparison

**Fig. 14:**
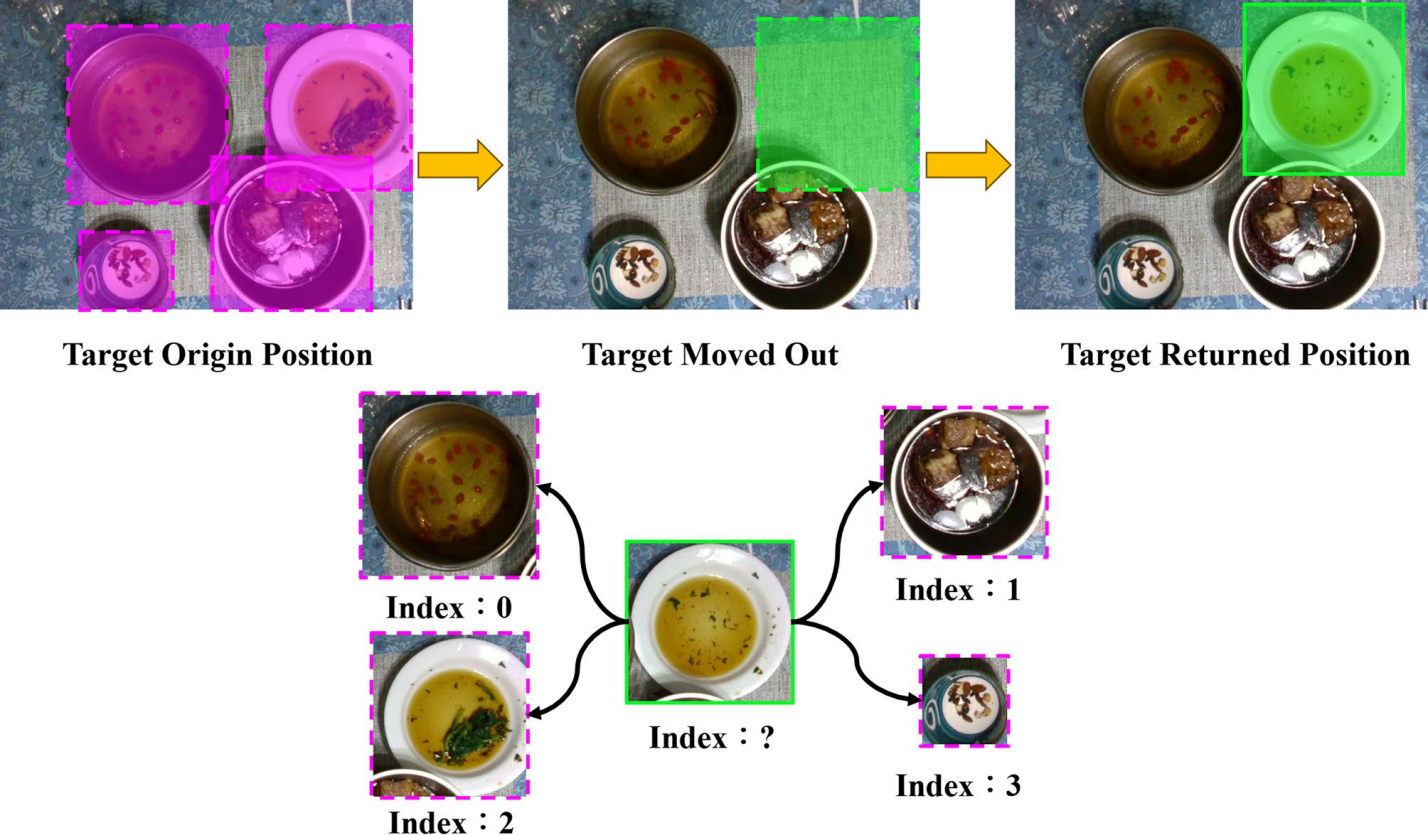
Workflow of association-based object matching.

Using the tracking algorithm, the system records the movement of plates in real time and detects when a plate is removed from the table. However, plates that leave the camera’s field of view cannot be continuously tracked. To address this limitation, a similarity measure is applied between plates that move off-screen and those still visible. The Hungarian algorithm is then used to optimize the matching results. The object association and matching process is illustrated in Fig. 14.

In this study, cosine similarity between plate features is calculated to determine final similarity. One plate’s data serves as the source, while data from four other plates are used as comparison targets. When processed by the Vision Transformer (ViT)^23^, each image is divided into RGB patches of a predefined size. Each patch is linearly embedded into a feature vector, producing a sequence of vectors that represent small image regions.

**Fig. 15:**
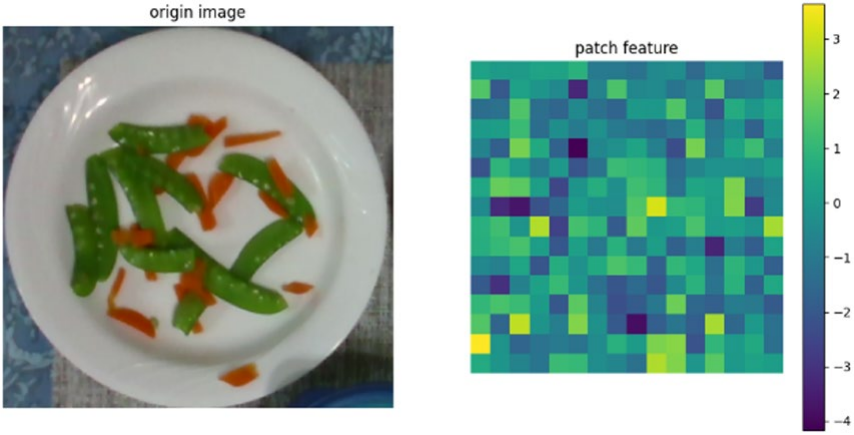
Feature Map of ViT.

These vectors are fed into the Transformer encoder, which contains multiple self-attention layers that model relationships between patches and extract comprehensive image features. As shown in Fig. 15, the left side presents the original plate image tracked by the algorithm, while the right side displays features extracted from one patch. After several encoder layers, the model outputs a feature tensor of size 1 × 197 × 768. Each 768-dimensional vector corresponds to the deep features of one patch. By compressing along the first dimension, all patch features are aggregated into a 1 × 768 vector, providing a compact representation of the entire image for comparison and downstream tasks.

This method enables the Transformer to capture both local details and global relationships, which is crucial for visual recognition. For plate analysis, this allows the model to consider the relative positions and interactions of items, thereby improving recognition accuracy.

**Fig. 16:**
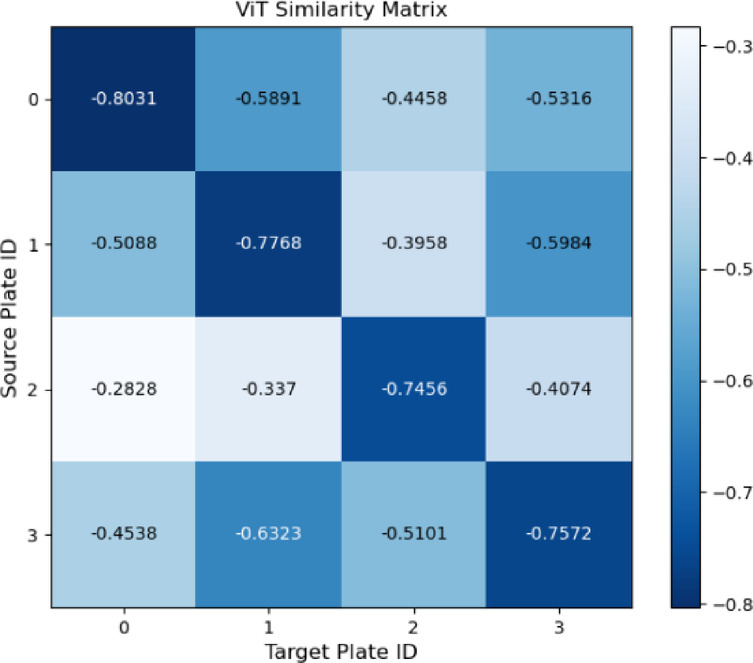
Comparison of feature similarities with ViT.

As shown in Fig. 16, the Vision Transformer network achieves high accuracy in similarity evaluation, demonstrating its advantage in image feature comparison tasks. However, its effectiveness may decline in scenarios where image features are less distinct. In future applications, combining multiple feature extraction methods could further improve matching accuracy. For example, incorporating circular radius similarity and computing RGB histograms for each channel (0–255 brightness range) can enhance results. Histogram similarity is then measured using the Bhattacharyya distance, enabling a more comprehensive analysis by integrating multiple features. When a balance between computational load and accuracy is required, the Vision Transformer alone provides an effective trade-off between performance and efficiency. Similar to other low-cost sensing systems that combine affordable hardware with machine learning for real-world deployment^24^, our method demonstrates how advanced feature extraction can be applied to practical dietary monitoring.

### Experimental results

**Fig. 17:**
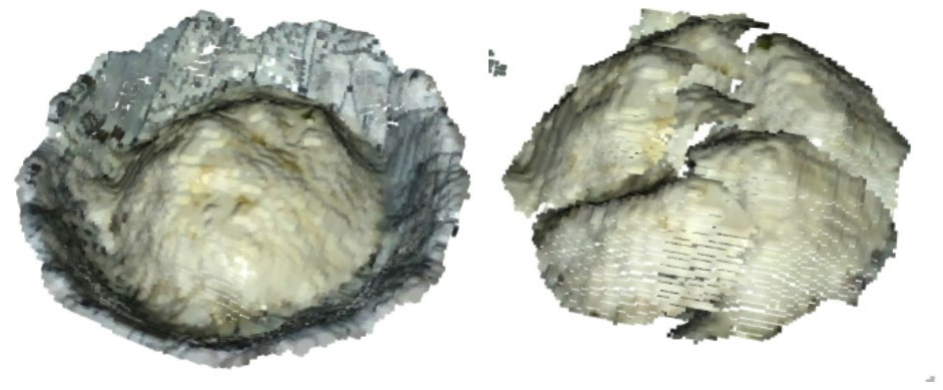
Point cloud segmentation of rice.

**Fig. 18:**
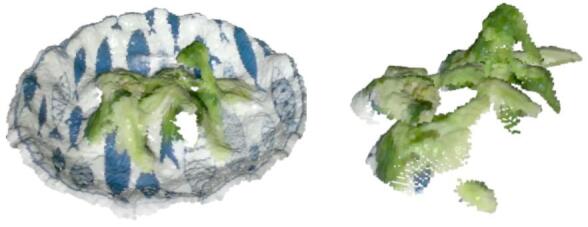
Point cloud segmentation of broccoli.

**Fig. 19:**
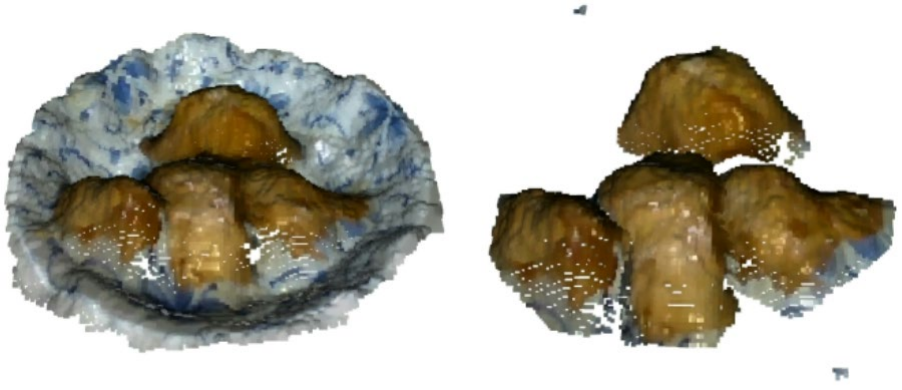
Point cloud segmentation of chicken.

Based on the modeling methods described in this study, three dishes—broccoli, chicken, and rice—were selected as experimental subjects. With the system’s sampling frequency set to one minute, food on the plate was segmented and integrated according to the user’s eating sequence, as shown in Figs. 17, 18 and 19.

By analyzing food volume at different time points, we examined consumption patterns throughout the meal. Figure 17 shows the segmentation of rice, Fig. 18 illustrates broccoli, and Fig. 19 presents chicken at various consumption stages.

These results reveal clear differences in consumption speed and patterns among different foods.

The collected data provide insights into eating habits and food consumption patterns, offering valuable references for nutritional recommendations. By calculating eating speed from sampled volume, the system can also analyze the nutritional composition of mixed-type foods.

Using the point cloud volume algorithm and Eq. (10) proposed in this study, and assuming food density equal to water, we tested three foods—broccoli, chicken, and rice—during actual meals. To evaluate whether the system could accurately accumulate weight during sequential eating, Table 1 reports the weight estimation for broccoli. The maximum error was − 30.68%, primarily due to the loose stacking of rigid foods such as broccoli, which are rarely cut into smaller pieces, resulting in overestimated point cloud volume.

Table 2 shows the results for chicken, with a maximum error of 26.35%, also caused by gaps in stacked portions. Table 3 presents the rice estimation, where the maximum error reached 30.34%. This was attributed to minimal differences between the point clouds in sequences 2 and 3, which caused part of the rice to be removed during non-overlapping segmentation, leading to underestimated weight. Overall, the average weight estimation errors across Tables 1, 2 and 3 were less than ± 10% when sequencing was not applied.

**Table 1:** Broccoli sequential eating weight Estimation.

Sequence	Absolute weight (g)	Volume (cm^3^)	Weight (g)	Error
1	11.5	18.3859	12.87013	−11.91%
2	14.5	17.3806	12.16642	16.09%
3	14	26.13619	18.295333	−30.68%
4	9.5	12.7545	8.92815	6.02%
5	12.3	15.8179	11.07253	9.98%
Total	61.8	90.4751	63.332563	−2.48%
Without Sequencing		93.9942	65.79594	−6.47%

**Table 2:** Chicken sequential eating weight Estimation.

Sequence	Absolute weight (g)	Volume (cm^3^)	Weight (g)	Error
1	30.8	30.4084	30.4084	1.27%
2	46.4	43.6534	43.6534	5.92%
3	47.3	34.8367	34.8367	26.35%
4	39.2	33.5642	33.5642	14.38%
Total	163.7	142.4627	142.4627	12.97%
Without Sequencing		155.6516	155.6516	4.92%

**Table 3:** Rice sequential eating weight Estimation.

Sequence	Absolute weight (g)	Volume (cm^3^)	Weight (g)	Error
1	36.5	28.6671	28.6671	21.46%
2	31.8	31.3682	31.3682	1.36%
3	39.7	39.8051	39.8051	−0.26%
4	23.8	16.5793	16.5793	30.34%
5	36.3	40.5053	40.5053	−11.58%
Total	168.1	156.925	156.925	6.65%
Without Sequencing		183.6207	183.6207	−9.23%

*Home deployment results*.

**Fig. 20:**
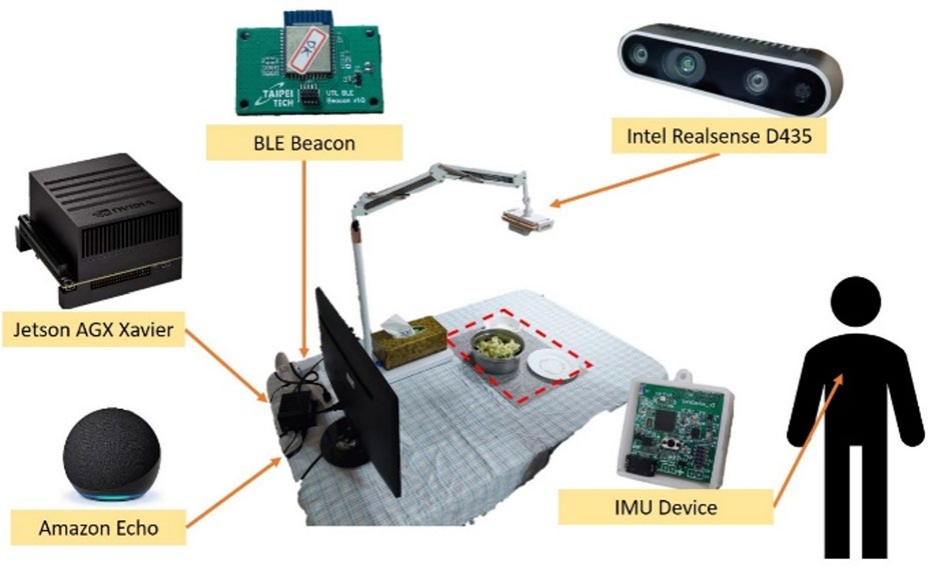
Experimental environment.

The experiments were conducted in a Linux environment using the RealSense D435 depth camera to collect depth point cloud data and RGB images. The camera was mounted 45–55 cm above the table in a horizontal, centered position. Lighting conditions were kept bright and stable, and the table surface was flat and uncluttered, as shown in Fig. 20. The camera sampled data at 30 FPS. The experimental procedure consisted of five steps: object recognition, object tracking, target association matching, food volume calculation, and nutritional value estimation based on food density.

To streamline the setup and reduce costs, the depth camera can be replaced with a standard webcam. As long as the viewing angle is consistent, food size can still be estimated. The Nvidia Jetson AGX Xavier can also be substituted with a Raspberry Pi 5, which uploads captured food images to the cloud. The chest-worn IMU device sends start and stop meal commands to the Raspberry Pi 5.

With this configuration, the total system cost is as follows: Raspberry Pi 5 (NTD 3,500) + Beacons (NTD 300 × 12 = 3,600) + Amulet (NTD 1,000 × 2 = 2,000) + Wristband (NTD 2,500 × 1) + Camera stand (NTD 1,000), totaling NTD 12,600.

**Fig. 21:**
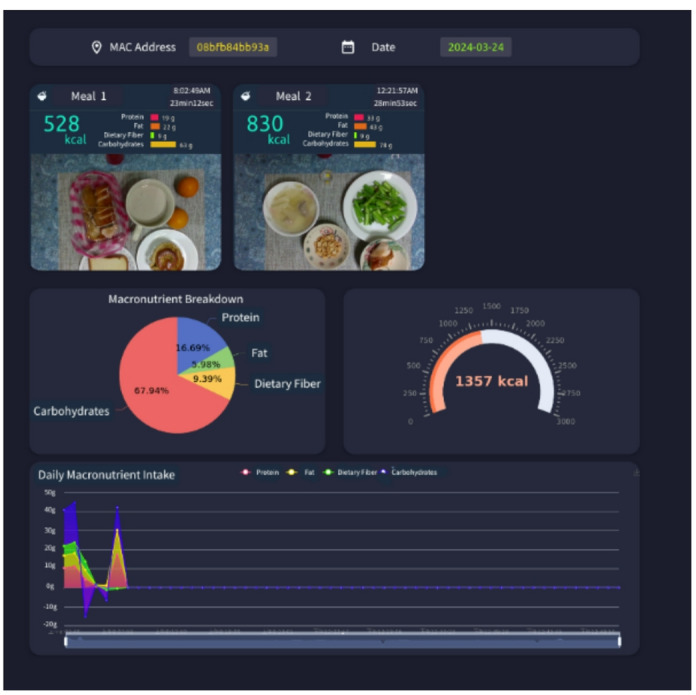
Subject’s dietary status of breakfast and lunch on 2024-03−24.

**Fig. 22:**
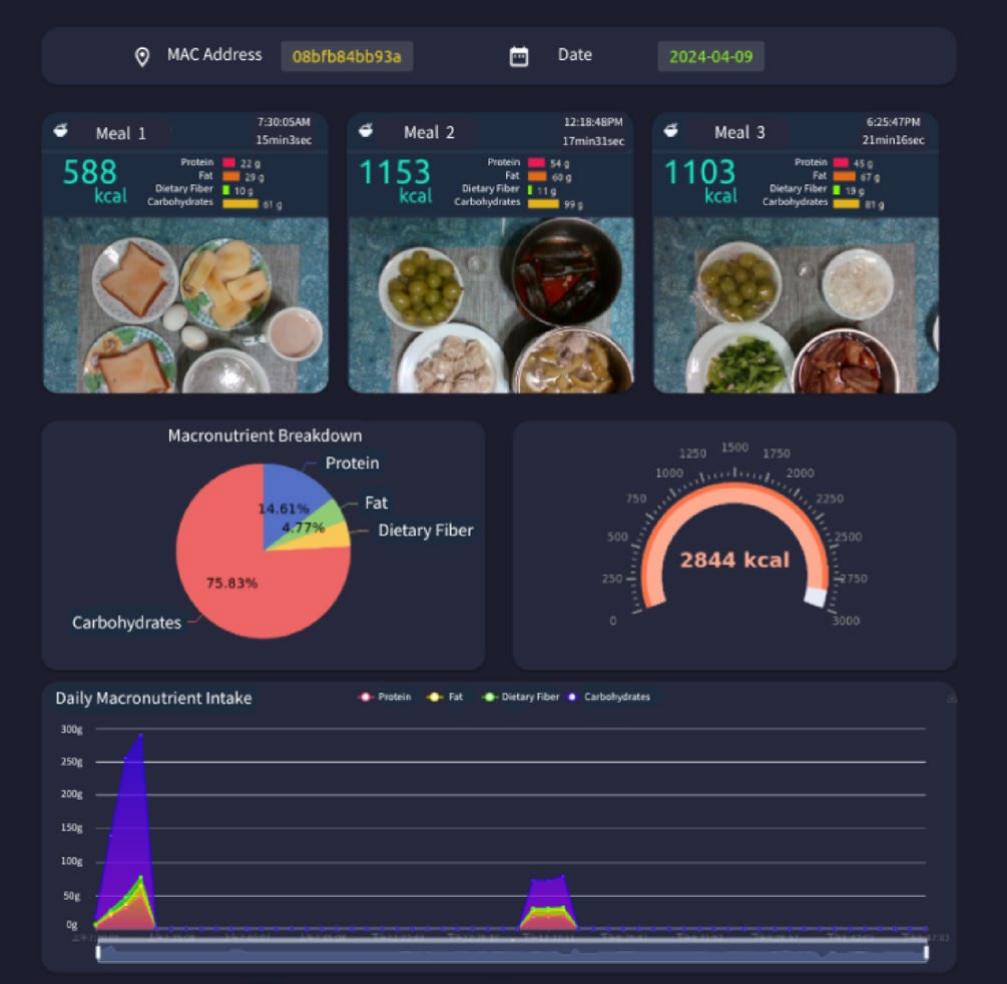
Subject’s dietary status of three meals on 2024-04−09.

The dietary assessment system proposed in this study was deployed in a test household consisting of a married couple, with nutritional calculations reflecting nearly three months of data for two individuals. The system uses the Nvidia AGX Jetson edge device for real-time computation and updates the MongoDB database. Users can preview daily meal data in real time, enabling dietary management and adjustments. The system also provides information on eating duration across different periods and the composition of nutritional elements, as illustrated in Figs. 21 and 22.

## Discussion and limitations

This paper proposes the integration of semantic segmentation and point cloud modeling to accurately document meal volume and composition, enabling comprehensive evaluation of home-based nutritional intake. Prior reviews^9^ summarize computing algorithms, mathematical models, and methodologies for image-based dietary assessment, comparing approaches for food recognition and volume/weight estimation in terms of speed, accuracy, efficiency, and constraints. To highlight the advantages and limitations of our method, Table 4 compares related studies by approach, technique, real-time detection capability, and performance.

Compared to previous studies, this work offers several key advancements. While other studies such as^25–27^, and^28^ also utilize RGBD cameras or vision-based methods to estimate food volume, they primarily focus on static and manual food image capture and detection. For instance, the work in^25^ uses 3D reconstruction with a reference card, achieving a volume estimation MAPE of 4.6%–11.1%. Similarly^26,27^, use RGBD cameras to estimate food weight and calories, respectively, but their methods also require manual intervention. The research in^28^ uses a mobile phone with depth sensors and deep learning to perform 3D reconstruction, but again, the process is static. The work in^29^ takes a different approach, using a GAN to estimate food energy, but it relies on a large dataset of static food images rather than live detection.

A major limitation of these existing approaches is the lack of real-time monitoring. Our method distinguishes itself by providing autonomous food image detection triggered by location-based IoT and wearable sensors, enabling real-time, live detection of meals. This is a significant improvement over the static and manual capture methods presented in the other studies. Our approach not only estimates food volume and calories using semantic segmentation and point cloud modeling but also provides unique features, such as diet speed detection and real-time meal detection. This allows for a more comprehensive and dynamic assessment of dietary intake in a real-world, home-based setting. While our average weight estimation errors for three types of food are less than ± 10%—a performance comparable to or even better than some of the methods in the literature (e.g^25,28^., —the true advantage lies in our method’s ability to provide continuous, automated monitoring without user intervention.

Ultimately, while the cited works contribute valuable methods for food volume and calorie estimation, they fall short in providing a seamless, real-time solution for continuous dietary monitoring. This work represents the first research to offer live detection of the volume and composition of meals, facilitating comprehensive and dynamic evaluations of real-time nutritional intake in a home environment.

However, when applying the method proposed in this study for food point cloud segmentation, the following applicability and limitation issues arise:

### The plate or container must not be made of flexible materials

**Fig. 23:**
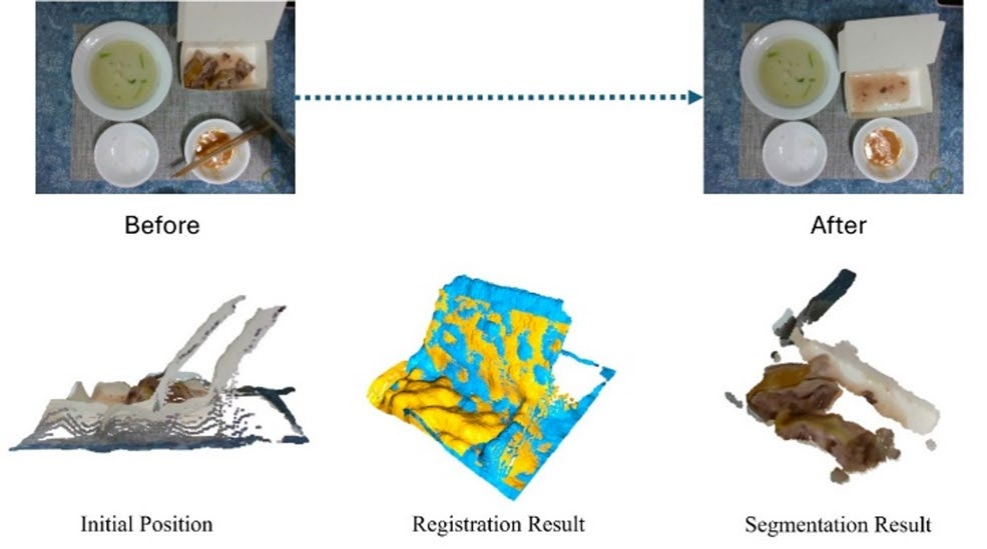
Paper meal box registration and segmentation results.

Flexible material containers, such as paper meal boxes, may deform due to handling or movement, which affects the accuracy of point cloud registration and segmentation. As shown in Fig. 23, when searching for non-overlapping point cloud areas, the point cloud data from a paper meal box may differ over time because it does not maintain a fixed shape like ceramic tableware. This deformation can interfere with registration and segmentation, resulting in the inclusion of non-food point cloud data in the segmentation, ultimately leading to calculation errors.

**Table 4:** Comparison with the related literatures.

	Approach	Technique	Live detection during meal	Performance
^26^	Motion 3D reconstruction with a reference card	Feature matching and pose estimation. Stereo matching and 3D reconstruction. Scale determination and volume estimation.	Static and manual food image capture and detection	The volume estimation’s MAPE for seven food types was 4.6%−11.1%
^27^	OAK-D Lite RGBD camera	Estimate food weight from images using advanced segmentation with a manually defined reference point (RP) and volumetric estimation.	Static and manual food image capture and detection	Average error of 2.86% for only rotisserie chicken images
^28^	RGBD camera	Capture an RGB-D food image. Estimate the food volume on the dish. Calculate calories using the pre-registered calorie density of each food category.	Static and manual food image capture and detection	Higher accuracy than CalorieCam and AR CalorieCam V2 application
^29^	Vision based method using real-time 3D reconstruction and deep learning view synthesis	A mobile phone with depth sensors captures a single depth image. A fine-tuned Mask R-CNN segments the food items. The depth image is converted into camera coordinates. The partial point cloud is processed by a point completion network (UNet) to perform 3D reconstruction. The portion size of food items is estimated.	Static and manual food image capture and detection	The average error for volume estimation ranged from 15–79 cm³ for eleven types of food. The U-Net model improved accuracy from 71.54% to 84.68%
^30^	Estimate food energy based on learned energy distribution images	This method uses a GAN to generate energy distribution maps from food images. A CNN thenpredicts food energy from these maps. This two-step process enables accurate food energy estimation.	Static and manual food image capture and detection	Average food energy estimation error 209Kcal for 347 food images which collected from the 45 community- dwelling men and women
This work	Depth camera RGBD Realsense	Take a RGBD food image Estimate volume of the food on the dish by semantic segmentation and point cloud modeling techniques Calculate foods calories using the pre-registered calorie density of each food category	Autonomous food image detection triggered by location based IoT	Average errors of weight estimation for three types of food are less than

### The plate or container must not be made of transparent materials

**Fig. 24:**
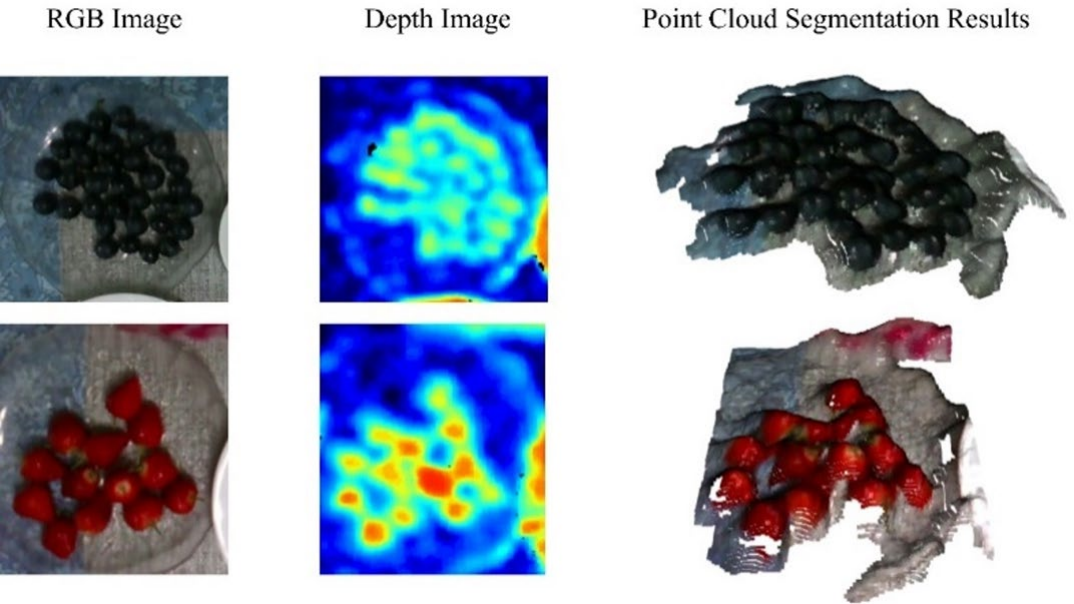
Depth information of transparent container.

Structured light depth cameras cannot capture depth information from transparent materials because projected light patterns do not reflect properly from their surfaces. As shown in Fig. 24, the transparent container is visible in the RGB image but nearly indistinguishable from the background in the depth map. In the point cloud, its appearance features are also absent. This results in registration errors, since the most stable alignment features normally come from the container’s exterior. When these features are lost, large changes in food shape cause registration to fail, making it impossible to identify matching points.

### Volume error for clear soup-type foods

**Fig. 25:**
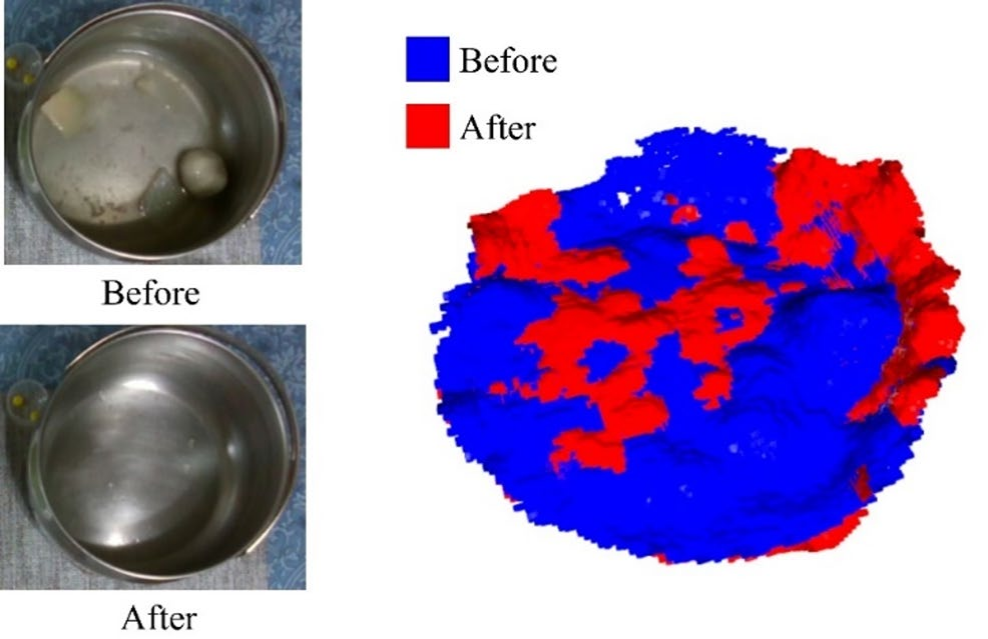
Depth comparison of clear soup-type foods.

Similar to transparent containers, structured light depth sensing causes light patterns to pass through transparent liquids and project onto the container’s bottom surface. As shown in Fig. 25, the point cloud height remains unchanged after the soup is removed. Consequently, for clear soups, the system cannot detect volume changes accurately, making nutritional analysis unfeasible.

## Conclusion

This paper proposes a dietary assessment system designed for home environments and adaptable to diverse dining habits. The system tracks food consumption by accounting for arbitrary plate placement and handling cases where bowls leave the camera’s view. Using point cloud segmentation, registration, and clustering, it models food with a maximum volume estimation error of 12.97%. A key advantage is its ability to capture internal food composition through repeated sampling. With semantic segmentation, it produces detailed mixed-nutrition profiles. Compared with the TFDA nutrition database, the maximum error for dietary fiber is approximately 28.55%, while errors for other nutrients (protein, fat, carbohydrates, and calories) remain within 20%.

In household deployment, the system simplified meal recording while accurately documenting meal times and uploading data to the cloud. Users could review eating habits via a web interface and adjust their dietary strategies accordingly. Through IoT integration, the system enabled interactive control via smart speakers and automatically triggered recording using indoor positioning Beacons and IMU posture detection, improving compliance and supporting consistent dietary records.

Nevertheless, the system showed limitations with flexible or transparent containers, where accurate volume estimation was not possible. Likewise, for clear soups, liquid volume could not be reliably measured, making nutritional value calculations uncertain.

### Future work

Beyond integrating the chest-worn IMU device and Beacon developed by our lab to monitor daily routines, the future direction of this research is also to incorporate wearable physiological devices like the Apple Watch, which can measure activity levels and provide hypertension alerts. We also aim to integrate Continuous Glucose Monitoring Systems (CGMS) to assist patients with chronic conditions, such as diabetes and chronic kidney disease, with their personal health management. By collecting diet content/speed, activity and weight/BMI, we may suggest personalized intervention: Tailored diet plans (e.g., specific food recommendations based on blood sugar response, adjustments to eating speed) and activity suggestions to prevent the progression to full-blown Type 2 diabetes or cardiovascular disease. Similarly the future study may apply to chronic kidney disease (CKD) management (especially in the early stages/complications of diabetes/hypertension), because CKD is often a complication of diabetes and hypertension. Diet (protein, sodium, potassium, phosphorus), hydration, and blood pressure control are crucial.

## Data Availability

The datasets used or analysed during the current study available from the corresponding author on reasonable request.
